# Mortality after infection with methicillin-resistant *Staphylococcus aureus *(MRSA) diagnosed in the community

**DOI:** 10.1186/1741-7015-6-2

**Published:** 2008-01-31

**Authors:** JA 'Chris' Delaney, Verena Schneider-Lindner, Paul Brassard, Samy Suissa

**Affiliations:** 1 Division of Clinical Epidemiology, McGill University Health Center, Royal Victoria Hospital, 687 Pine Avenue West, Ross 4.29, Montreal, H3A 1A1, Canada; 2 Department of Epidemiology, Biostatistics and Occupational Health, McGill University, Montreal, Canada

## Abstract

**Background:**

Outbreak reports suggest that community-acquired methicillin-resistant *Staphylococcus aureus *(MRSA) infections can be life-threatening. We conducted a population based cohort study to assess the magnitude of mortality associated with MRSA infections diagnosed in the community.

**Methods:**

We used the United Kingdom's General Practice Research Database (GPRD) to form a cohort of all patients with MRSA diagnosed in the community from 2001 through 2004 and up to ten patients without an MRSA diagnosis. The latter were frequency-matched with the MRSA patients on age, GPRD practice and diagnosis date. All patients were older than 18 years, had no hospitalization in the 2 years prior to cohort entry and medical history information of at least 2 years prior to cohort entry. The cohort was followed up for 1 year and all deaths and hospitalizations were identified. Hazard ratios of all-cause mortality were estimated using the Cox proportional hazards model adjusted for patient characteristics.

**Results:**

The cohort included 1439 patients diagnosed with MRSA and 14,090 patients with no MRSA diagnosis. Mean age at cohort entry was 70 years in both groups, while co-morbid conditions were more prevalent in the patients with MRSA. Within 1 year, 21.8% of MRSA patients died as compared with 5.0% of non-MRSA patients. The risk of death was increased in patients diagnosed with MRSA in the community (adjusted hazard ratio 4.1; 95% confidence interval: 3.5–4.7).

**Conclusion:**

MRSA infections diagnosed in the community are associated with significant mortality in the year after diagnosis.

## Background

Methicillin-resistant *Staphylococcus aureus *(MRSA) is increasingly implicated in potentially lethal hospital-acquired infections [1,2]. Some of the MRSA strains that are found in patients in the community have been acquired during hospitalization or a visit to a hospital. However, MRSA strains likely originating outside of hospitals and different from those found in hospitals have been identified [3-5]. People who are infected by MRSA in the community have different risk factors than those infected in hospitals [1,2], particularly as common risk factors in hospitals may be rare or absent in the community.

While hospital-acquired MRSA infections can be fatal [1,2], cases of severe and life-threatening MRSA infections from the community have also been reported [6-8] and these case reports suggest that the prognosis of community-acquired MRSA infections may be poor [9]. However, hospital-based studies do not capture patients from the community whose infection does not require hospital care, and thus such studies or case reports cannot evaluate the impact of this infection on mortality. To date, no population-based study has evaluated the prognosis and mortality of MRSA patients diagnosed in the community.

We thus conducted a large cohort study to assess mortality associated with MRSA infections diagnosed in the community in comparison with a population-based sample of patients free of this infection.

## Methods

We used the General Practice Research Database (GPRD) between 1 January 2001 and 31 December 2005 for this study. The GPRD contains the diagnostic, testing and prescribing records of approximately 3.2 million patients from over 400 general practices in the United Kingdom (UK) as recorded by their GPs. The GPRD is extensively used for medical research [10,11] and has been used previously for prognostic work in cardiovascular disease [12], as well as infectious disease research [13,14]. GPRD data is routinely audited for quality [10] and is considered to be of high validity for medical research.

### Cohort definition and follow-up

We conducted a frequency-matched cohort study. The cohort consisted of all patients with MRSA diagnosed between 1 January 2001 and 31 December 2004, and a population-based sample of patients without MRSA. We defined MRSA to be the diagnosis 'Methicillin-resistant *Staphylococcus aureus *positive' (READ code 4JP..00) entered into the patient's medical record. The date of the MRSA diagnosis was the MRSA patient's cohort entry date. For each patient with MRSA, we randomly selected up to 10 patients free of MRSA that were registered in the GPRD at their corresponding MRSA patient's cohort entry date and that were matching on practice, cohort entry date and age (± 2 years). We assigned to these MRSA-free patients their corresponding MRSA patient's cohort entry date. Patients of the cohort, both with and without MRSA, had to be at least 18 years of age at their cohort entry date, had been registered in the GPRD for at least 2 years prior to their cohort entry date and had no hospitalizations recorded in the GPRD in the 2 years before cohort entry date. Frequency matching on cohort entry date is particularly important to control for calendar time effects, because the rate of MRSA diagnosis in the GPRD was not constant over the time period of our study [13]. This time variation in risk could have been a potential source of bias in our estimates of effect [15]. By frequency matching on practice we also ensured that patients with and without an MRSA diagnosis came from the same geographic region of the UK, thus indirectly controlling for factors such as socio-economic status and geographical variations in disease frequency.

Follow-up was from day 1 to day 365 after cohort entry date. Patients were censored if they transferred out of practice for a reason other than death or were free of the outcome 365 days after cohort entry. Patients who died on day 0 were excluded. It was possible for a patient without MRSA to be diagnosed with MRSA during follow-up.

### Outcome

The outcome, death, was defined as a transfer out of the GPRD with the reason being 'Death' recorded in the patient database of the GPRD. We used the date of the transfer out as the date of death. As the GPRD is based on GP records and deaths are a variable that practices in the GPRD are audited on [10], death should be a well-recorded variable in this database.

For a secondary outcome, to assess the level of morbidity caused by MRSA infections diagnosed in the community, we considered a composite outcome of either death or hospitalization. For this outcome, the date of outcome was either the date of hospitalization or the date of death; whichever came first. The recording of reason for hospitalization is poor in the GPRD, but the hospital visit itself is well recorded [10,11]. Therefore, we were not able to separate hospitalization by reason and used 'any cause' hospitalization. The recording of hospitalization information also made it impossible to assess whether or not patients diagnosed with MRSA had serious infectious complications such as bacteraemia.

### Covariates

In addition to age and sex, we defined a series of baseline covariates that could have an impact on the prognosis of individuals diagnosed with MRSA in the community. We included the presence of cancer, heart disease, renal failure, autoimmune diseases and diabetes, all identified using medical records in the GPRD in the 2 years prior to cohort entry date. We also considered use of antibiotics and oral prednisone in the year before cohort entry date.

### Statistical analysis

We used the Kaplan-Meier method to estimate the cumulative mortality in patients with and without MRSA over the one-year follow-up. The Cox proportional hazards model was used to estimate the hazard ratio of death associated with MRSA infection, adjusting for covariates [16]. Proportionality of hazards was assessed using graphical methods.

We conducted several sensitivity analyses. First, to determine the maximum possible effect of censoring on the results, we used both worst-case and best-case imputation, appropriate techniques because of the small number of censored observations [17]. For worst-case imputation, we assumed that all MRSA patients who were censored die immediately and that all patients without MRSA lived until the end of follow-up. For best-case imputation, we assumed that all censored MRSA patients lived until the end of follow-up and all censored MRSA-free patients died immediately. Second, as an additional control for overall health status, we considered past antibiotic use as a marker of susceptibility to infection. Third, we determined the effect of MRSA diagnosis among patients with none of the co-morbid conditions in our study to estimate the effect of MRSA among healthy patients. We used SAS version 9.1.3 in all analyses.

We obtained ethics approval for this study from the Scientific and Ethical Advisory Group of the GPRD and the McGill University Health Center Research Ethics Board.

## Results

**Figure 1:**
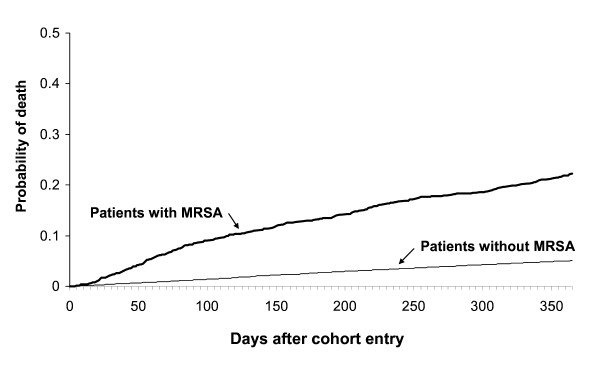
**Kaplan-Meier plot of the cumulative probability of death up to 1 year in patients after diagnosis with MRSA by a GP and patients without MRSA matched on diagnosis date, age and general practice**. Data from the GPRD (2001–2004).

The cohort included 1439 patients diagnosed with MRSA between 2001 and 2004, and 14,090 matching disease-free patients from the GPRD. Over the one-year period after cohort entry, 21.8% of patients diagnosed with MRSA and 5.0% of those without the diagnosis died. The Kaplan-Meier plot is shown in Figure 1. The loss to follow up owing to transfer out of practice was comparable in the patients with and without MRSA (4.2% and 3.0%, respectively). Patients with MRSA diagnosis were more likely to be male and to have co-morbid conditions predictive of mortality as compared with patients without a diagnosis of MRSA (Table 1).

**Table 1:** Baseline characteristics of patients diagnosed with MRSA (1439 subjects; mean ± SD age 70.3 ± 18.3 years) and a matched population sample free of MRSA (14,090 subjects; mean ± SD age 69.9 ± 18.2 years) from GPRD (2001–2004)

**Characteristic**	**Patients with MRSA**		**Matched MRSA-free patients**	
	
	*n*	Percentage	*n*	Percentage
Male	604	42.0%	5988	42.5%

Female	835	58.0%	8102	57.5%

Co-morbidity in the 2 years prior to cohort entry				

Diabetes	162	11.3%	877	6.2%
Cardiovascular disease	98	6.8%	467	3.3%
Stroke	74	5.1%	140	1.0%
Peripheral vascular disease	34	2.4%	72	0.5%
COPD	76	5.3%	364	2.6%
Renal failure	45	3.1%	138	1.0%
Cancer	27	1.9%	127	0.9%
Autoimmune disease	12	0.8%	66	0.5%
Drug exposure in the 1 year prior to cohort entry				
Oral prednisone use	155	10.8%	858	6.1%
Antibiotics	1019	70.8%	5369	38.1%

Patients with a diagnosis of MRSA were more likely to die than those without MRSA (unadjusted hazard ratio (HR), 4.85; 95% confidence interval (CI), 4.25–5.54; see Table 2). After adjustment for age, sex and the co-morbid conditions listed in Table 1, the HR of death from any cause in patients with MRSA compared with patients without MRSA was 4.08 (95% CI: 3.54–4.69).

**Table 2:** All-cause mortality and hospitalization or all-cause mortality within a year in patients diagnosed with MRSA compared with matched patients free of MRSA from the GPRD (2001–2004) The hazard ratio is adjusted for all variables in Table 1. Death or hospitalization patients were followed until first event of either kind and only counted once.

**Characteristic**	***n***	**Events**	**Person-years of follow-up**	**Rate of event per 100 years**	**Crude HR**	**Adjusted HR (95% CI)**	
**Death**							

Patients with MRSA	1439	313	1224	25.6	4.85	4.08	(3.54–4.69)
Patients without MRSA	14,090	709	13510	5.2	1.00	1.00	(Reference)

**Death or hospitalization**							

Patients with MRSA	1439	485	1106	43.9	3.93	3.31	(2.97–3.69)
Patients without MRSA	14090	1440	13106	10.2	1.00	1.00	(Reference)

**Figure 2:**
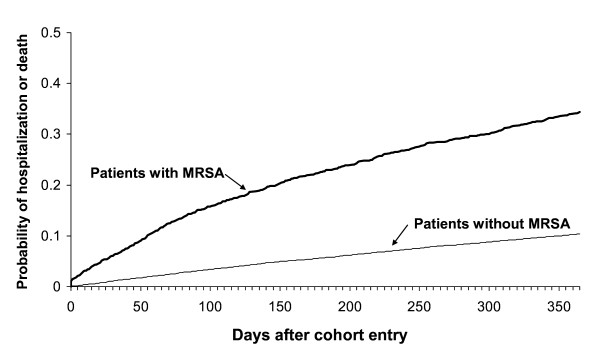
**Kaplan-Meier plot of the cumulative probability of hospitalization or death in patients up to 1 year after diagnosis with MRSA by a GP and patients without MRSA, matched on diagnosis date, age and general practice**. Data from the GPRD (2001–2004).

The cumulative incidence of the composite outcome of death or hospitalization is depicted by the Kaplan-Meier plots in Figure 2. Table 2 shows that the adjusted HR of this outcome with MRSA infection is 3.31 (95% CI: 2.97–3.69).

The sensitivity analysis for informative censoring produced an adjusted HR of 4.91 (95% CI: 4.30–5.61) for worst-case imputation, and an adjusted HR of 2.69 (95% CI: 2.36–3.07) for best-case imputation. Prior antibiotic use had a small effect on mortality once we adjusted for other factors with an adjusted HR of 1.30 (95% CI: 1.14–1.49). Finally, among the subset of patients with no co-morbidities, the effect of MRSA infection on mortality was comparable to that obtained for all patients with an adjusted HR of 4.86 (95% CI: 4.10–5.77).

## Discussion

In this study, we demonstrated an increase in mortality within a year of a diagnosis with an MRSA infection in the community. This increased mortality cannot be completely explained by underlying co-morbid conditions as we adjusted for the major risk factors of death. The four-fold increase in mortality persisted across several sensitivity analyses. In addition, we found a risk increase of similar magnitude with the combined outcome of death and hospitalization.

Patients with nosocomial MRSA bacteraemia [18,19] have been reported as having as great as a ten-fold increased risk of death [18]. However, these extremely severe infections are not representative of all nosocomial MRSA infections, and likely not representative of community-acquired MRSA infections, many of which do not require hospitalization [6]. As expected, our observed death rate is much lower than in these studies.

In this study we observe an average of 360 MRSA infections diagnosed in the community per year among the adult GPRD population during the study period. This is about 13 cases per 100,000 adults in the GPRD population, which is similar to the incidence found in other studies such as that by Fridkin et al [4]. This is especially true if we assume that some MRSA infections are diagnosed in hospital emergency rooms and thus not counted in our study. Fridkin et al also found a significant proportion of community-associated MRSA infections occurring in patients above 65 years of age [4].

In this study we considered a large cohort of patients diagnosed with MRSA infections in the community and a large group of matching disease-free patients all selected from a database that is representative of the UK population [10]. The death rate among the disease-free patients is broadly consistent for this age and sex distribution with that seen in UK vital statistics [20]. Using the GPRD allows us to study patients with all levels of disease severity encountered by GPs, including those not treated in hospitals and thus not part of any hospital-based investigation. The study design (frequency matched cohort study) ensured that the distribution of key covariates (general practice, age and calendar time) was balanced between patients with and without MRSA at baseline. By matching on general practice we indirectly matched on unmeasured factors such as socio-economic status, area of residence and nursing home care, which are usually common to the patient population of a given general practice. These demographic factors have been shown to be important in the epidemiology of MRSA [21]. By matching for practice we control for the potential confounding nature of these variables.

There are nevertheless some limitations to our study. In particular, we could not distinguish site or severity of individual MRSA infections. As we could not examine antibiotics given in hospital, it was not feasible to use post-diagnosis treatment either to separate infection site or infection source as different therapy is recommended for community-acquired MRSA [22]. However, our observed mortality of MRSA infections diagnosed by general practitioners in the community rules out that these are the extremely severe infections seen in hospitals [18]. On the other hand, these results imply that individuals with MRSA diagnoses in the GPRD are not mostly asymptomatic carriers but include clinically significant infections: a conclusion supported by other work in the GPRD on MRSA infections [13]. Moreover, we strongly believe that currently, general practitioners in the UK do not routinely screen and thus diagnose MRSA in asymptomatic individuals in the community.

We could not exclude the possibility that some MRSA infections were acquired in hospitals and not in the community, because we had no information on potential transmission from visits to or work in hospitals. We could not look at the spread of MRSA from close contacts [23] or clusters in workplaces or schools [24]. Therefore, we describe here the prognosis of MRSA infections detected by general practitioners, but we cannot demonstrate that these infections arose independently in the community. However, the high mortality rate underlines the importance of their control. To contribute to achieving this, we recently proposed the appropriate use of antibiotics [13].

The increased mortality observed for MRSA infections may be a result of a health state that predisposes to MRSA infections or residual confounding by this health state. Frailty and residing in a nursing home are examples of potential confounders that we were unable to adjust for in this study. If the mortality associated with MRSA is a manifestation of underlying susceptibility to infections, then past antibiotic use could serve as an estimate of the degree of bias in our study. Past antibiotic use should not be associated with distant outcomes but will show patients who have a history of infections. While we found a small increase in mortality predicted by antibiotic prescriptions given in the year prior to cohort entry, it was much too small to be a major confounder of the observed increase in mortality. Therefore, some degree of susceptibility to infection may explain a small part of the increased death rate. However, because of the small size of this effect we can infer that the risk of death in this study is not principally a marker of an underlying susceptibility to infections.

We did not report on the effect of antibiotic therapy on mortality after MRSA diagnosis for three reasons. One, the GPRD does not report drug exposures during hospitalization. This would result in the most severe cases of MRSA infection being misclassified as unexposed. This could lead to a falsely protective effect of antibiotic use if it was only recorded among the healthiest patients. Two, the use of antibiotics would be a dynamic treatment regime and more advanced statistical methods would be required to obtain a valid estimate [25]. Three, the GPRD recording system does not allow us to determine whether an antibiotic prescription is being given for the original infection or for a second infection that developed independently.

While previous work in this database has shown differences between classes of antibiotics and the development of MRSA diagnosed in the community [13], we did not see any strong link between antibiotic class and prognosis. Therefore, we considered antibiotics only as a single drug class for this analysis as the principal reason for including antibiotics as a covariate was to control for possible differences in susceptibility to infections between patients with and without MRSA.

The broad use of sensitivity analysis [26,27] is a key strength of our study. The results of these analyses indicate that the increase in mortality after a diagnosis of MRSA is robust to changes in assumptions. The use of the READ code 4JP..00 for MRSA infection has been discussed previously [13] and patients with this code were more similar in characteristics to post-operative MRSA wound infections than to carriers. The very high mortality rate seen in patients with MRSA in the present study, independent of baseline health status, is also highly suggestive of infections rather than carrier status. However, carrier status may be associated with lower mortality and hospitalization rate in comparison to clinically relevant infections. Thus, including this code may have led to an underestimation of the impact of a diagnosis of MRSA in the community on hospitalization and mortality.

The largest remaining source of possible bias in this study is the possibility of unmeasured confounding or, more likely, residual confounding. While non-parametric approaches could be used to reduce residual confounding in this study, they are unlikely to explain effects of the size that we observe [28]. It is not likely that such large effects could be explained by anything other than many extremely strong confounders [29] based on estimates of the effect of confounding. We also avoided introducing bias from either the misallocation of person time [30] or from adjusting for variables potentially in the causal pathway between MRSA infection and mortality [31] by defining confounders using only the baseline characteristics of the subjects. Adjusting for factors (such as secondary infections) that arise after the diagnosis of MRSA infection could lead to overadjustment [32] and could bias the estimates towards the null.

## Conclusion

In conclusion, our study suggests that MRSA can be a potentially serious infection in the community leading to increased mortality. Further research will allow a better understanding of MRSA diagnosed in the community and the prognosis of patients with this diagnosis. This is essential for effective prevention of MRSA arisen from the community, which will not only reduce hospital admissions with infections, spread into and within hospitals, and transmission in the community, but also likely will prevent deaths in the community.

## Competing interests

The author(s) declare that they have no competing interests.

## Authors' contributions

All authors contributed to the conception and design of the article, along with the acquisition and interpretation of the data. JACD and VSL performed the statistical analysis and drafted the manuscript. All authors were involved in revising the article for important intellectual content and approved the final version to be published.

## Pre-publication history

The pre-publication history for this paper can be accessed here:


